# Role of oxytocin in bone

**DOI:** 10.3389/fendo.2024.1450007

**Published:** 2024-09-03

**Authors:** Tianming Wang, Jianya Ye, Yongqiang Zhang, Jiayi Li, Tianxiao Yang, Yufeng Wang, Xiao Jiang, Qingqiang Yao

**Affiliations:** ^1^ Department of Orthopedic Surgery, Institute of Digital Medicine, Nanjing First Hospital, Nanjing Medical University, Nanjing, China; ^2^ Department of Orthopedic Surgery, Huaian Hospital of Huaian City, Huaian, China

**Keywords:** bone metabolism, oxytocin, BMSC, osteoblast, osteoclast, chondrocyte, estrogen, osteoporosis

## Abstract

Oxytocin (OT) is a posterior pituitary hormone that, in addition to its role in regulating childbirth and lactation, also exerts direct regulatory effects on the skeleton through peripheral OT and oxytocin receptor (OTR). Bone marrow mesenchymal stem cells (BMSCs), osteoblasts (OB), osteoclasts (OC), chondrocytes, and adipocytes all express OT and OTR. OT upregulates RUNX2, BMP2, ALP, and OCN, thereby enhancing the activity of BMSCs and promoting their differentiation towards OB rather than adipocytes. OT also directly regulates OPG/RANKL to inhibit adipocyte generation, increase the expression of SOX9 and COMP, and enhance chondrocyte differentiation. OB can secrete OT, exerting influence on the surrounding environment through autocrine and paracrine mechanisms. OT directly increases OC formation through the NκB/MAP kinase signaling pathway, inhibits osteoclast proliferation by triggering cytoplasmic Ca2+ release and nitric oxide synthesis, and has a dual regulatory effect on OCs. Under the stimulation of estrogen, OB synthesizes OT, amplifying the biological effects of estrogen and OT. Mediated by estrogen, the OT/OTR forms a feedforward loop with OB. Apart from estrogen, OT also interacts with arginine vasopressin (AVP), prostaglandins (PGE2), leptin, and adiponectin to regulate bone metabolism. This review summarizes recent research on the regulation of bone metabolism by OT and OTR, aiming to provide insights into their clinical applications and further research.

## Introduction

Almost all vertebrate express oxytocin or oxytocin-like genes and translated peptides (1). Oxytocin (OT) is a nonapeptide hormone composed of cysteine, tyrosine, isoleucine, glutamine, aspartic acid, proline, leucine, glycine (Cys–Tyr–Ile–Gln–Asn–Cys–Pro–Leu–Gly) with a relative molecular mass of 1007 (2). Isoleucine needs to be at the third position in the structure to stimulate the oxytocin receptor (OTR) and exert its function (3). A disulfide bridge between Cys residues 1 and 6 is linked by a disulfide bond (4). This divides it into two parts, one consisting of a ring portion of six amino acids and the other of the C-terminal portion of three amino acids (5). Both parts have biological effects of the hormone.

There are two forms of OT in the human body, central OT and peripheral OT. Central OT is synthesized and produced by the paraventricular nucleus (PVN) and supraoptic nucleus (SON) of the hypothalamus, and released into the bloodstream by the neurohypophysis under appropriate stimulation (6). Central OT has anti-inflammatory, antioxidant, energy metabolism regulation, increased confidence, enhanced social engagement, and alleviation of conditions such as autism, depression, schizophrenia, and obsessive-compulsive disorder (7–10). There is relatively limited research on the relationship between central OT and the musculoskeletal system, with no breakthrough discoveries to date. The relationship between central OT and bone metabolism remains unclear. Peripheral OT is produced by tissues such as adipocytes, osteoblasts, uterus, ovaries, and testes, and plays a direct role in cardiovascular system and bone homeostasis, apart from its involvement in breastfeeding and parturition (11–13). Clinical studies have shown that postmenopausal women with osteoporosis have significantly lower plasma oxytocin levels than non-osteoporotic patients, and being in a state of low oxytocin levels can further exacerbate bone loss in postmenopausal osteoporotic women (14). Studies have shown that the trabecular bone volume of OT and OTR gene knockout mice is significantly reduced, and injection of oxytocin into their peritoneum can effectively reverse bone formation defects caused by decreased osteoblast differentiation (15). Similarly, intraperitoneal injection of OT in ovariectomized rats can significantly reduce the decrease in osteoblasts, increase in osteoclast numbers, decrease in serum OPG/RANKL ratio, and increase in bone formation marker levels (16). This further demonstrates the promoting effect of peripheral OT on bone synthesis metabolism. Numerous studies have found that OT can promote fracture healing, increase bone mass, protect cartilage, alleviate pain, and improve mood and mental state, among other positive effects. This has great prospects for application in orthopedics. This article will focus on reviewing the relationship between OT and bone metabolism.

## Timeline of oxytocin discoveries

**Figure 1:**
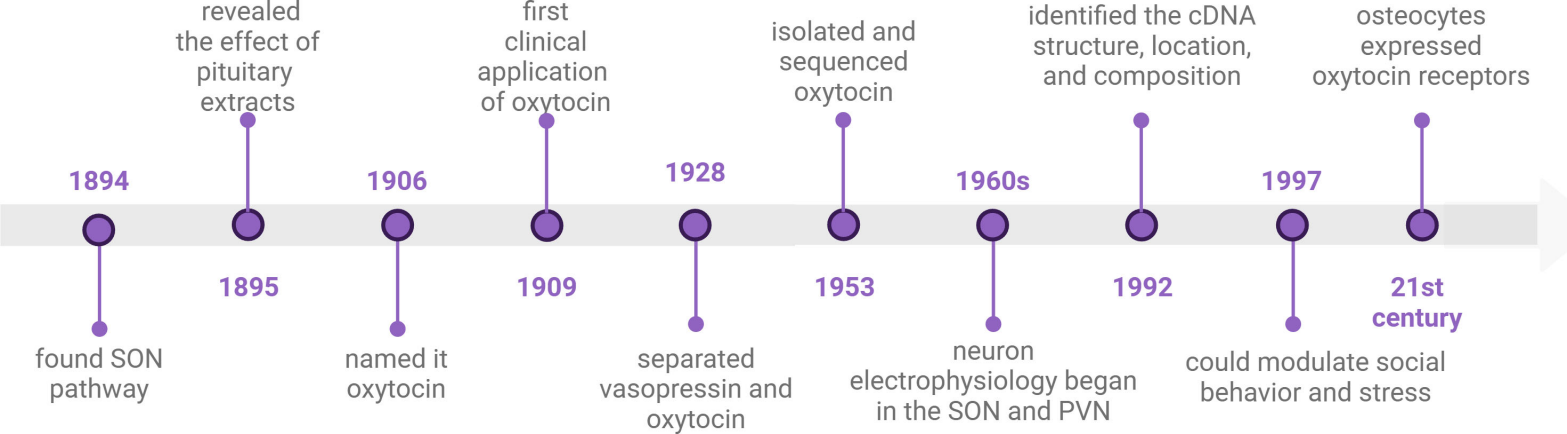
timeline of oxytocin discoveries. This illustration created with BioRender (https://biorender.com).

In 1894, S. Ramón y Cajal described the neuronal pathway from the supraoptic nucleus (SON) to the posterior pituitary lobe. Building upon this, G. Oliver and E. A. Schäfer in 1895 first elucidated the physiological effects of the pituitary and its extracts. A decade later, British researcher Henry H. Dale christened the pituitary extract from the early pregnant cat uterus as oxytocin due to its uterine contraction properties (17). At that time, scholars widely held the view that the pituitary extract contained solely oxytocin without other constituents. In 1909, Canadian W. Blair Bell proposed the idea of using oxytocin in obstetric labor to shorten the duration of labor (18). This marked the commencement of the clinical application of OT. It was not until 1928 that O. Kamm employed a dialysis membrane to segregate vasopressin and OT, thereby dispelling the notion that the pituitary extract consisted solely of OT (19). Subsequently, OT remained in the phase of natural extraction. Approximately two decades later, Vincent du Vigneaud isolated OT from the freeze-dried posterior pituitary of cattle and successfully sequenced it for the first time (20, 21). The sequencing results expedited the transition from the natural extraction of OT to its artificial synthesis (22). Following this, researchers embarked on studying the effects of OT and endeavored to identify the associated receptors. Scientists discovered that OTRs are not only present in peripheral tissues but also in the central nervous system. In 1992, Kimura et al. delineated the cDNA structure, location, and composition of the human OTR (23). Subsequently, Copland JA, Ives KL, and others identified OTR expression on human osteoblasts and stimulated PGE2 synthesis in bone trabecular cells (24). OT and PGE2 are implicated in osteoporosis, with PGE2 known to foster bone formation (25). PGE2 has been demonstrated to be beneficial for bone formation. OT is a novel bone metabolic agent. Intranasal administration of OT can modulate social behavior and stress (23). Subsequent research unveiled the crucial role of oxytocin in social psychological disorders such as anorexia nervosa, autism, and anxiety (7, 26, 27). In the 21st century, the widespread presence of OTRs in peripheral tissues, including adipocytes, and the existence of OTR in human osteoclasts indicate that OT also regulates osteoclasts (28) (**Figure 1**).

Over the ensuing decade, scientists discovered that OT can ameliorate osteoporosis, and osteoarthritis, and promote periodontal bone regeneration. In 2014, Park JW conducted research on the osteogenic effects of loaded OT scaffolds, paving the way for novel applications of OT in tissue engineering technology (29).

## Oxytocin receptor

The human oxytocin receptor (OTR) single-copy gene is located on chromosome 3p35-3p26.2 (4). The OTR belongs to the G protein-coupled receptor superfamily, consisting of a polypeptide with 389 amino acids (30). It is composed of seven hydrophobic transmembrane domains, an extracellular N-terminus, and an intracellular C-terminus, with alternating intracellular and extracellular loops connected by alpha helices (31). This structure is highly conserved in the G protein-coupled receptor superfamily. Conserved residues in GPCRs may be involved in a common mechanism for activating and signal transducing G proteins. Activation of the OTR may involve, similar to adrenergic receptors, the opening of a solvent-exposed site in the cytoplasmic domain that participates in G protein recognition (32, 33).

The OTR can convert extracellular stimuli (neurotransmitter or hormone release) into intracellular signaling events (cellular functions and regulatory changes). The OTR is coupled to a GTP-binding protein, which, together with a GTPase, stimulates phospholipase C-β isoform activity. This leads to the production of inositol trisphosphate and 1,2-diacylglycerol. Inositol trisphosphate triggers intracellular calcium release, while diacylglycerol activates protein kinase C, phosphorylating unidentified target proteins. Ultimately, in response to the increase in intracellular Ca2+, various cellular events are initiated. This process is accompanied by transient or sustained ERK phosphorylation. In smooth muscle cells, the Ca2+-calmodulin system activates myosin light chain kinase activity, initiating smooth muscle contraction, such as in uterine smooth muscle. In neurosecretory cells, elevated Ca2+ levels control cell excitability, regulate their firing patterns, and lead to neurotransmitter release, such as in taste perception (31, 34).

## Direct effect of oxytocin: regulating bone metabolism cells

OTRs are present in both peripheral and central systems, including the brain, reproductive system, mammary gland tissues, cardiovascular system, kidneys, adrenal glands, thymus, pancreas, adipocytes, and periodontal tissues (35). Studies indicate that OTRs are present in various bone cells, including osteoblasts (OBs) and osteoclasts (OCs). This suggests that OT directly interacts with bone cells, influencing their function and activity, and regulating bone formation and adipogenesis (36).

### Oxytocin regulates BMSCs

**Figure 2:**
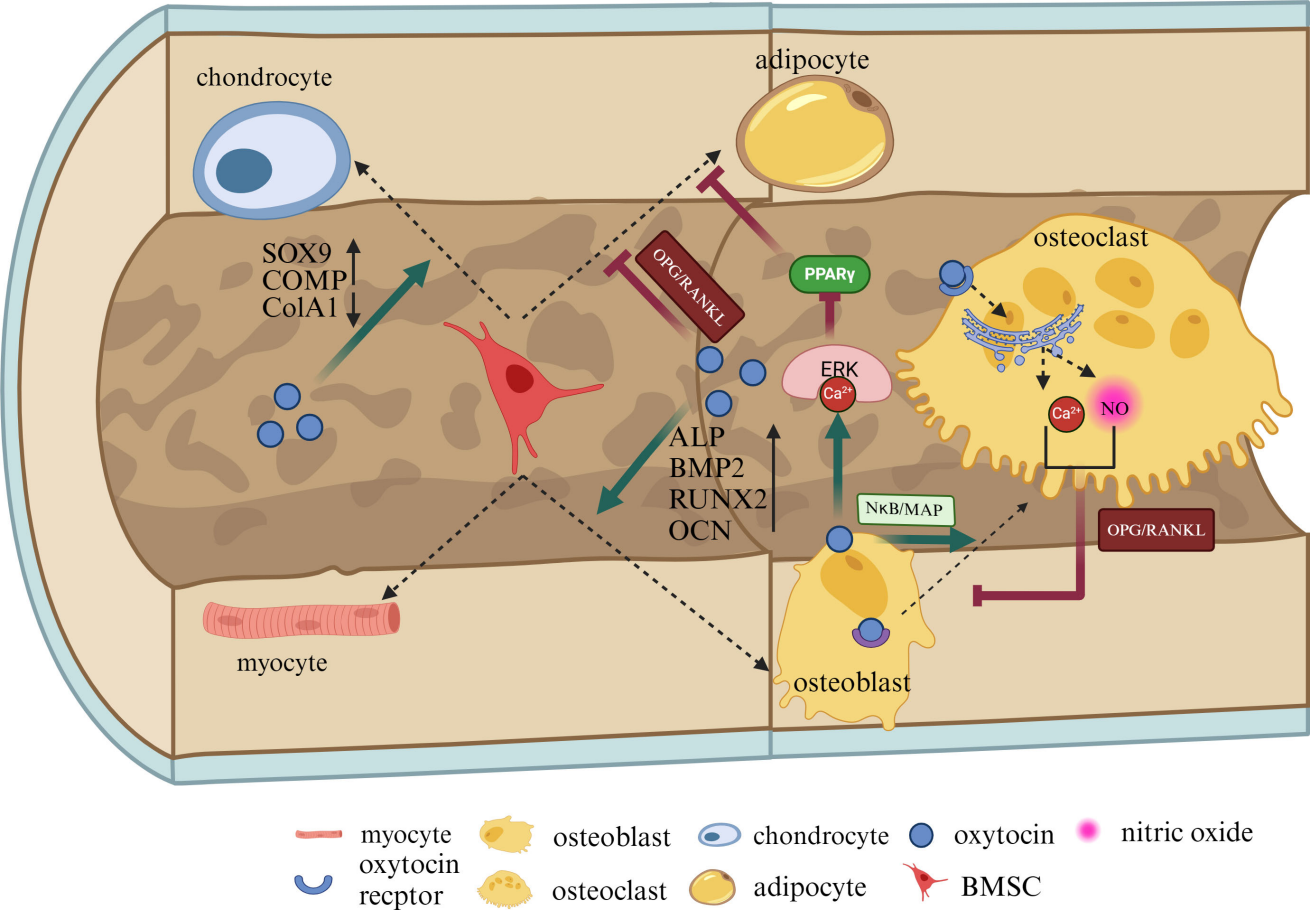
OT can directly regulate BMSCs, chondrocytes, adipocytes, and osteoblasts. OT promotes the proliferation of BMSCs and their differentiation into OBs, increasing ALP, BMP-2, OPN, and OCN. OBs can secrete OT themselves, exerting influences on the surrounding environment in both autocrine and paracrine forms. OT can directly increase OCt formation by the NκB/MAP kinase signaling pathway. After stimulating mature OCs, OT can inhibit OCs by leading to the release of nitric oxide and calcium ions (Ca2+) from the endoplasmic reticulum. Additionally, the Ca2+-Erk1/2 pathway phosphorylates peroxisome proliferator-activated receptor gamma (PPARγ), thereby reducing adipogenesis. OT can directly regulate OPG/RANKL to inhibit adipocyte production, increase the expression of SOX9 and COMP, reduce the expression of ColA1, and protect cartilage. This illustration created with BioRender (https://biorender.com).

BMSCs, known as bone marrow stromal cells, possess the potential to differentiate into OBs, adipocytes, chondrocytes, and myocytes (37). Research by Fallahnezhad et al. has shown that OT upregulates the expression of proteins such as OPG, RUNX2, and OCN, increases the extracellular levels of insulin-like growth factor 1 and alkaline phosphatase (ALP), enhances the cell viability of BMSCs derived from ovariectomized rats, and strengthens their potential to differentiate towards OBs (38). Studies by Santos et al. indicate that OT, in combination with osteogenic inducers, significantly increases the content of OT and OTR in BMSCs from adult and aged female rats (39). OT can promote the proliferation of BMSCs and their differentiation into OBs, enhancing ALP activity and increasing the gene expression of BMP-2, OPN, and OCN (**Figure 2**) (40). These studies demonstrate that OT plays a role in promoting the proliferation of BMSCs, enhancing their activity, inducing their differentiation towards OBs, and mineralization, which may be crucial for increasing bone density.

### Oxytocin regulates osteoblasts

Osteoblast (OBs) are primarily responsible for synthesizing and secreting bone matrix and mineralizing the bone, playing a crucial role in bone formation. OBs and adipocytes differentiate from the same mesenchymal cell precursors. During the differentiation process, OT is produced by OBs in the bone marrow and acts as a paracrine-autocrine regulator of bone formation, with high expression of OTR in OBs (41). The binding of OT and OTR leads to the release of calcium ions (Ca2+) from the endoplasmic reticulum, activating the Ca2+-Erk1/2 pathway. The Ca2+-Erk1/2 pathway enhances osteogenic effects by increasing the expression of the osteogenic gene RUNX2 (**Figure 2**). The Ca2+-Erk1/2 pathway also phosphorylates peroxisome proliferator-activated receptor gamma (PPARγ), thereby reducing adipogenesis (31, 42).

The direct and predominant role of OT in bone homeostasis is mainly achieved through stimulating OB formation and bidirectionally regulating OC generation (30). Studies have shown that at the cellular level, OBs from OT and OTR knockout mice exhibit lower mineralization activity and significant downregulation of key genes representing OB differentiation, such as OCN, RUNX2, OPN, etc. (43). BinGe et al. found that OT activates the Erk and protein kinase B (AKT) pathways (44). At a concentration of 50nM OT, the expression of osteogenic-related genes is upregulated in protein imprinting, including ALP, type I collagen (Col I), Runx2, OPN, and OCN (44).

Research by the Tamma team found that after OT intervention, OT can stimulate ALP activity in osteoblasts upregulate BMP-2 through the Schnurri-2, and activate transcription factor 4 (ATF4) pathways, thereby promoting OB differentiation into a mineralized phenotype (45). Other studies suggest that *in vivo*, OT activates the C-FOS pathway and upregulates BMP-2. BMP-2 controls the expression of Schnurri-2 and 3, osterix, and ATF4, regulating osteoblastogenesis (46).

By intraperitoneal injection of OT in rats, it has been verified that OT has anti-osteoporotic effects, reducing the risk of femoral neck fractures. The mechanism is related to increasing the number of bone cells and OBs, regulating levels of bone-specific alkaline phosphatase (bALP), osteocalcin, tartrate-resistant acid phosphatase (TRAP), and the expression of the Osterix/Sp7 transcription factor OPG/RANKL pathway (47). Research by Di et al. found that under the stimulation of OT, OTR promotes the translocation of OTR to the nucleus of osteoblasts through continuous interactions with beta-negative regulatory factors, small GTPase Rab5 protein, nuclear transport protein beta-1, and transport protein-1, promoting osteoblast differentiation and mineralization by increasing ALP activity and regulating the OPG/RANKL-related pathways (43). Therefore, OT promotes OBs to synthesize and secrete bone matrix and mineralize the bone, accelerating bone regeneration.

### Oxytocin regulates osteoclasts

Osteoclasts (OCs)originate from the mononuclear-macrophage system and are primarily responsible for resorbing organic and mineral components of the bone matrix, removing old and damaged bone tissue, and playing a crucial role in bone development, growth, repair, and remodeling (48). OCs express high levels of OTRs (30). In mature OBs, OT lowers the level of osteoprotegerin (OPG), stimulates the expression of receptor activator of nuclear factor kappa-Β ligand (RANKL) in OBs, and induces OC differentiation (49). OT has a bidirectional regulatory effect on OCs. On one hand, it directly increases OC formation by activating NκB, mitogen-activated protein kinase (MAPK) signaling pathways, and mitogen-activated protein kinase signaling, and indirectly increases OC formation by upregulating RANKL. On the other hand, after stimulating mature OCs, OT can inhibit bone resorption by triggering cytoplasmic Ca2+ release and nitric oxide synthesis (45, 50, 51). Studies have shown that after OT treatment, the number of OCs in the bone marrow of ovariectomized mice significantly decreases, while bone density levels significantly increase. This mechanism may involve restoring the coupling of OBs or OCs in the body by altering the ratio of RANKL to OPG (52). In conclusion, OCs express OT and OTR, and OT exerts a dual regulatory effect on OCs by triggering cytoplasmic Ca2+ release and nitric oxide synthesis, adjusting the ratio of OPG/RANKL in OBs, thus both inhibiting and promoting OC activity (**Figure 2**).

The expression of OTRS in pre-osteoclasts is lower than in mature OCs, suggesting that the level of OTR expression may be related to different stages of OC differentiation (28). Treatment with OT leads to increased proliferation of pre-osteoclasts, indicating that OT may be involved in controlling the early stages of OC differentiation and formation.

### Oxytocin regulates chondrocytes

Chondrocytes are differentiated from BMSCs and are capable of generating and maintaining cartilage matrix, primarily composed of collagen and proteoglycans (53). Normal cartilage development is crucial for bone formation during endochondral ossification, and any abnormalities in the microenvironment of the bone matrix can lead to the development of osteoarthritis (OA) (54). OT is expressed in chondrocytes and acts on subchondral bone, stimulating cartilage formation by binding to OTRs (55). Studies have shown a significant correlation between low levels of OT and OA in patients, with decreased expression of OT in chondrocytes of OA patients and a dose-dependent response to tumor necrosis factor-α treatment, while administration of OT has potential beneficial effects on cartilage, subchondral bone, muscle, and inflammation (56). OT has the potential to maintain cartilage integrity. Furthermore, research by Wu et al. has found that OT reverses the gene and protein expression of MMP-1 and MMP-13 in chondrocytes in a dose-dependent manner through the Janus kinase 2/signal transducer and activator of transcription 1 (JAK2/STAT1) pathway, and gene knockout of OTR eliminates the inhibitory effect of OT on MMP-1 and MMP-13 (55). These studies suggest that chondrocytes also express OT and stimulate cartilage formation by binding to OTR. Additionally, OT prevents cartilage matrix degradation by regulating matrix metalloproteinases and inhibits bone immune inflammatory responses, which may be key to improving bone microstructure. In a previous study by Roux CH, OT induced an increase in the expression of aggregating proteoglycans, cartilage oligomeric matrix protein (COMP), and SRY-related HMG-box gene 9 (Sox9) after culturing chondrocytes, while downregulating the expression of the fibrous tissue marker ColA1 and increasing the content of glycosaminoglycans in the extracellular environment, indicating that OT can promote chondrocyte generation (**Figure 2**). Additionally, OT mitigates the detrimental effects of IL-1β, reduces ADAMTS-4 mRNA transcription levels, and plays a protective role in alleviating inflammation in cartilage (57). These findings demonstrate that OT influences the function of chondrocytes and can protect articular cartilage.

### Oxytocin regulates adipocytes

In physiological conditions, BMSCs possess equal differentiation potential towards adipocytes and osteoblasts. However, in pathological conditions, regulatory mechanisms favoring either osteogenesis or adipogenesis can weaken the ability of MSCs to differentiate in the opposite direction (58). Recent studies have revealed that adipocytes also express RANKL/OPG and play a crucial role in influencing osteoclast development and regulating bone remodeling (59). The differentiation of BMSCs towards adipocytes is a key event in the pathogenesis of osteoporosis (OP) (60). OT inhibits the differentiation of adipocyte precursors by regulating the receptor activator of the NκB ligand/OPG axis signaling pathway, thereby reversing bone loss and bone marrow adiposity (16).

Culturing human multipotent adipose-derived stem cells with varying concentrations of OT has been shown to enhance osteogenic effects and the activity of SA-β-galactosidase (61). This suggests that OT promotes osteogenic differentiation in a dose-dependent manner and may enhance osteogenesis in human adipose-derived stem cells (hASCs) by modulating the autophagy process. The intrinsic connection between adipocyte differentiation and bone metabolism influences the differentiation or function of bone tissue cells. OT also stimulates OB differentiation and inhibits adipocyte differentiation through the OPG/RANKL signaling pathway, thereby determining bone formation over adipogenesis (62).

## Indirect effect of oxytocin

### Oxytocin and estrogen

**Figure 3:**
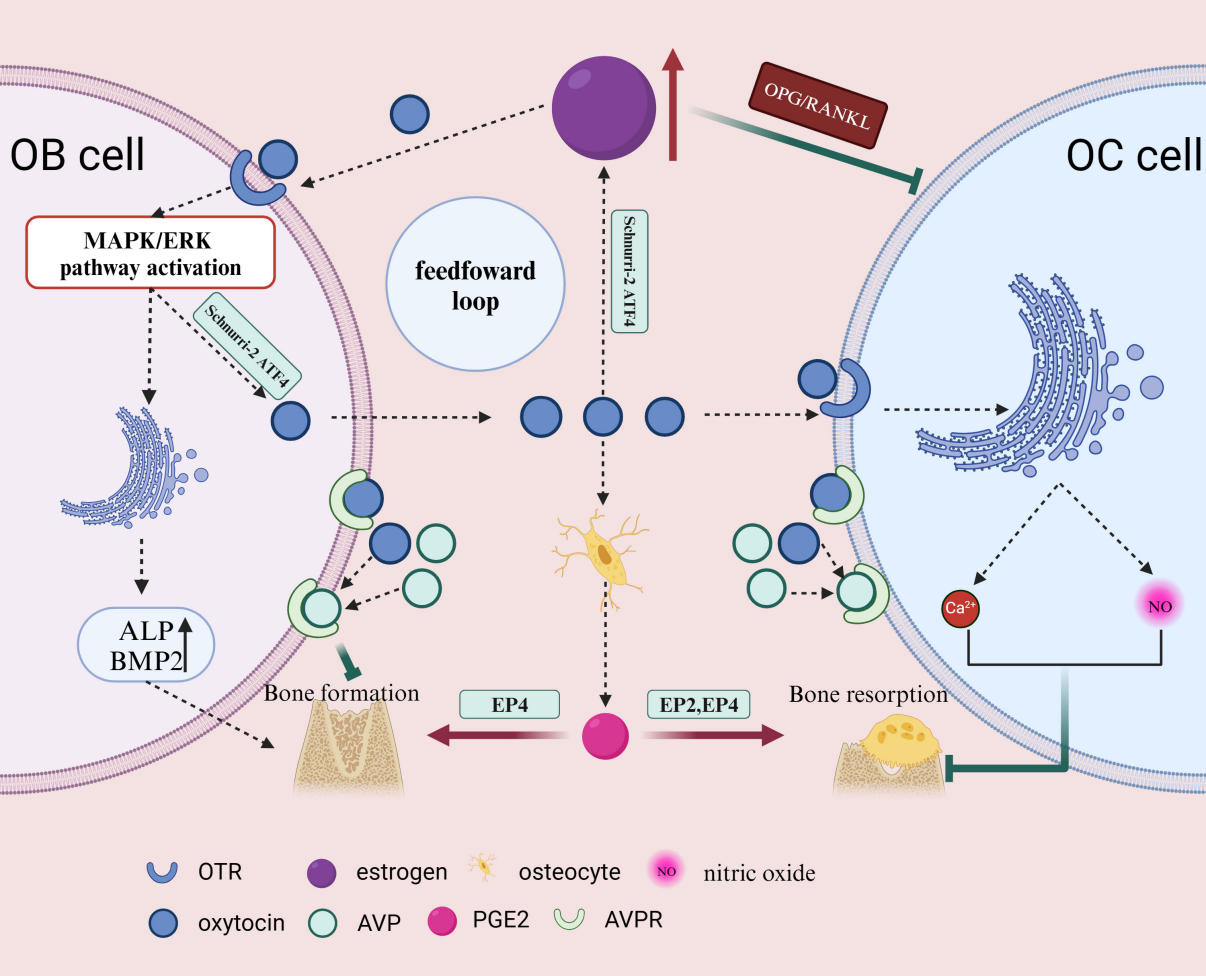
Estrogen inhibits OC bone resorption through the OPG/RANKL pathway and can stimulate bone formation at higher doses. OB, when influenced by estrogen, typically reactively produces OT, which further amplifies the effects of estrogen, increases OT production, and forms an estrogen-mediated OT positive feedback loop. AVPR-1a and -2a are expressed in OBs and bone cells. OT and AVP compete for the same receptors on OB, exerting opposite effects. OT can also stimulate bone cells to secrete prostaglandins, promote OB differentiation, and enhance osteogenic ability. OT can induce PGE2 synthesis in bone cells, and PGE2 regulates bone formation and resorption through EP2 and EP4 receptors. After stimulating mature OC, OT can inhibit OC by triggering intracellular Ca2+ release and nitric oxide synthesis. This illustration created with BioRender (https://biorender.com).

Estrogen exerts its effects on bone through two mechanisms: it can inhibit bone resorption by OCs through the OPG/RANKL pathway and stimulate bone formation at higher doses (49, 63, 64)(**Figure 3**). Estrogen also regulates OBs, bone cells, and T cells to modulate OC differentiation and activity, thereby inhibiting bone resorption and promoting bone formation (65). Studies have shown that the absence or silencing of OTR in OBs inhibits estrogen-induced OB differentiation. *In vivo*, OTR knockout mice do not exhibit the increased trabecular bone volume, cortical thickness, and bone formation induced by estrogen (64). During pregnancy or lactation when estrogen levels are elevated, OT release in the bone marrow promotes rapid bone recovery and inhibits bone resorption. OTR in OBs is essential for estrogen’s actions (66). Further research has revealed that after being influenced by estrogen, OBs typically produce OT in response, and OT, upon binding with its receptor OTR on OBs, amplifies the effects of estrogen, increasing OT production and forming an estrogen-mediated OT/OTR feedforward loop (40). The OT/OTR feedforward loop OT comprising input factors OT and estrogen, output factor OTR, and the signaling pathway Schnurri-2/ATF4, represents a novel regulatory cycle for bone metabolism. Local synthesis of OT by OBs is estrogen-mediated. 17β-estradiol stimulates OT production through the Erk phosphorylation pathway (67). In summary, estrogen mediates the synthesis of OT and its receptor. OT serves as a synthetic metabolic mediator for estrogen’s effects on the skeleton.

### Oxytocin and arginine vasopressin

The gene for OT is located on the human chromosome 20, and the same is true for arginine vasopressin (AVP) (6). The distance between OT and AVP on the human chromosome is approximately 8kb (7). Being on the same chromosome, they may have some common actions in the body. AVPR-1a and -2a are expressed in OBs and bone cells (36) (**Figure 3**). Although AVPR-2 is also expressed in OBs and OCs, it does not affect bone metabolism (68). OT and AVP share high-affinity G protein-coupled receptors in bone cells, but they have opposite effects on the skeleton (69). AVP and OT can partially share receptors. In mice with both AVPR-1a and OTR deletions, the increased bone mass and upregulated osteogenic gene expression are attenuated, indicating that OTR plays a dominant role (36). While OTR is not essential for the inhibitory effects of AVP on OB generation and gene expression, when OTR is absent in AVPR-1a knockout cells, gene expression stimulated by AVP is suppressed. This suggests that AVP and OT can share their receptors in regulating bone formation in OBs (68).

### Oxytocin and PGE2

OT can promote the synthesis of prostaglandin E2 (PGE2) in human Bone trabecular cells (24). OBs participate in the regulation of sensory nerves by activating the PGE2 receptor 4 (EP4) and inhibiting sympathetic nerve activity through the central nervous system. The PGE2-EP4 sensory nerve axis regulates neural activity and mesenchymal stem cell differentiation in the bone marrow of adult mice, promoting bone formation and inhibiting fat production by inhibiting sympathetic activity in the central nervous system (70, 71) (**Figure 3**). PGE2 can also stimulate BMSC osteogenic differentiation through the endothelial cell-mediated ERK pathway (72). While PGE2 has a promoting effect on OCs, the specific mechanism is still unclear. This may be attributed to the activation of EP4 and EP2 receptors on OCs, affecting OC migration and function (73).

### Oxytocin and leptin, adiponectin

OT can also act on cytokines (such as leptin and adiponectin) to indirectly affect bone tissue (74). Leptin has two opposing effects on bone tissue. It mainly inhibits bone formation by restricting OB proliferation, while also stimulating OB differentiation and inhibiting OC activity (75). These two pathways complement each other. Adiponectin (APN) has the potential to regulate bone mass positively, stimulate angiogenesis, and inhibit OCs (76). Adiponectin can inhibit OC differentiation induced by macrophage colony-stimulating factor (M-CSF) and receptor activator for NκB ligand RANKL (77). The two receptors of APN are expressed in OBs, stimulating osteoblast differentiation and mineralization, and playing a direct role in bone metabolism (78).

## Oxytocin and bone related diseases

### Oxytocin and osteoporosis

Osteoporosis (OP) is a systemic bone disease characterized by loss of bone density and quality, disruption of bone microstructure, and increased bone fragility. Primary OP is associated with age-related decline in estrogen levels, particularly in postmenopausal women (79). Secondary OP can be caused by various factors such as chronic use of glucocorticoids (80). Ovariectomy is a commonly used model to simulate postmenopausal OP. The lack of estrogen leads to reduced trabecular perforation and connectivity loss in bone (81). Estrogen plays a protective role in cortical bone quality (82). This protection is mediated through estrogen receptors expressed in the bone cell lineage. Estrogen can induce the synthesis of OT in bone (83). In adipocytes, OT may convert estradiol to estrone by affecting the action of 17β-hydroxysteroid dehydrogenase, a steroid hormone enzyme (66). Estradiol amplifies the bone-forming effects of OT by stimulating OB. Estrogen must activate OT signaling to enhance its promoting effect on bone. Previous studies have shown that oxytocin (OT−/−) and oxytocin receptor (OTR−/−) knockout mice exhibit OP. Fallahnezhad established an ovariectomy model in adult female Wistar rats. They found that OT treatment could promote the late-stage mineralization of BMSCs induced by ovariectomy *in vitro* bone formation process while altering the expression of OCN, OPG, and Runx2 genes (40). Beranger GE et al. demonstrated that OT can reverse OP (16). The results showed that OT treatment restored bone parameters to normal in ovariectomized mice. OT treatment increased the RANKL: OPG ratio to reverse OP. This ratio directly regulates OBs and OCs *in vivo (* 52). Elabd C et al. support this view and add that OT can stimulate PGE2 synthesis, promoting bone formation and inhibiting adipocyte differentiation (41). Another possible pathway is OT activation of MEK1/2 and ERK1/2, affecting human BMSCs (5).

Postmenopausal women are more likely to have lower OT levels (14). Due to their decreased OT levels, they are more prone to OP and muscle atrophy (84). Supplementing with OT can increase bone density, regulate muscle metabolism, and delay the onset of muscle atrophy (85, 86). Endogenous OT plays an important role in peri-menopausal bone remodeling, while exogenous OT becomes a potential preventive intervention during this period to improve bone quality and functional outcomes, potentially providing better gait activity (87). OT can maintain joint stability and bone health in males (88).

Psychological stress may also be a contributing factor to OP. Excessive stress can accelerate bone loss and reduce bone formation (70). Psychological stress and OP share similar pathogenic mechanisms, including glucocorticoids, insulin-like growth factors, etc (89). OT can alleviate stress. Therefore, OT may delay OP by relieving stress. OT can induce lipolysis and inhibit the gene program for converting white fat to brown fat by expressing OTR in adipocytes (66, 90). Yi KJ et al. fed mice a high-fat diet and found that OT expression was associated with adipocyte differentiation and obesity accumulation, rather than fat content. This promotes the osteogenic effects of human multipotent adipose-derived stem cells and BMSCs (38, 90). In conclusion, OT can reduce fat generation, promote bone formation, and reverse OP (**Table 1**).

**Table 1:** Mechanism of OT and orthopedic diseases.

	Therapeutic effects	Mechanism
Osteoporosis (OP)	Increase bone mass Delay bone loss	Influence RANKL/OPG, regulating OBs and OCs in the body
Osteoarthritis (OA)	Protect cartilage Inhibit inflammation	Activated C-FOS/AP-1 and JAK2/STAT1 pathways, delayed cartilage degradation, inhibited the release of inflammatory cytokines
Fracture	Accelerate bone healing	Activate C-FOS and Ca2+-Erk pathways, promote bone formation, and inhibit bone resorption.
Analgesia	Alleviate pain Improve mood	Modulate peripheral nociceptors and dorsal root ganglia

### Oxytocin and osteoarthritis

**Figure 4:**
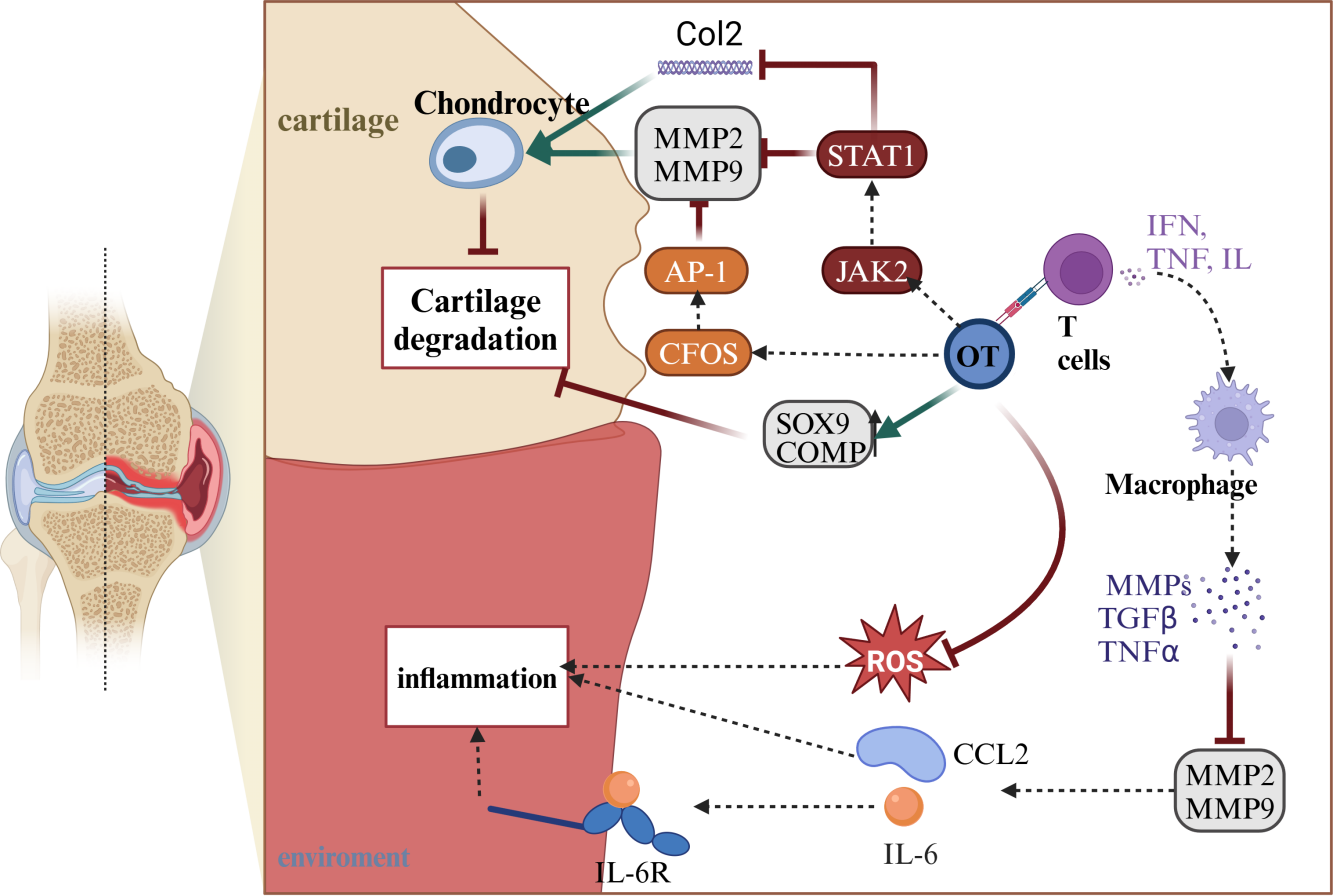
OT can indirectly reduce inflammatory responses by regulating MMP2 and MMP9 through T cells, as well as directly decreasing ROS to control inflammation. In the treatment of arthritis, OT can activate the CFOS/AP-1 pathway and STAT1/JAK2 pathway, which collectively act on MMP2 and MMP9 to alleviate cartilage degradation. The STAT1/JAK2 pathway can also increase the expression of Col2, protecting chondrocytes. Furthermore, OT can directly increase the protein content of SOX9 and COMP, promoting chondrocyte proliferation to protect cartilage and achieve the goal of treating arthritis. This illustration created with BioRender (https://biorender.com).

Osteoarthritis (OA) is a progressive degenerative joint disease characterized by cartilage destruction, subchondral changes, and osteophyte formation (91). It is associated with age, obesity, genetics, and significant joint injuries (91, 92). Patients with OA experience chronic pain in the absence of specific treatment. Within the body, glial cells, OT, and AVP systems are activated (93). Additionally, OA is associated with cartilage-degrading enzymes and inflammatory cytokines (55, 56) (**Figure 4**). The most crucial cartilage-degrading enzyme is matrix metalloproteinase (MMP), which includes inflammatory factors such as tumor necrosis factor-alpha (TNF-α), and TGF-β superfamily proteins. Among the MMP family, MMP-1 and MMP-13 are more influential than other members (84). T lymphocytes play a role in controlling metalloproteinase activity. Regulatory T cells are involved in downregulating MMP2 and MMP9 metalloproteinases, releasing pro-inflammatory cytokines such as CCL2 and IL-6 (94). OT and estrogen are related to T cells (95). There may be a connection between OT, T cells, and OA. The current mainstream view is that OT regulates T cell secretion of IFN, TNF, and IL families, affecting downstream macrophage activation of MMP, and slowing cartilage degradation (96). Exploring the specific effects of the oxytocin-T cell-OA axis is crucial. OTR is present in chondrocytes. OTR expression is reduced in OA patients (57). OT can inhibit the secretion of pro-inflammatory cytokines, including TNF-α, IL-6, and reactive oxygen species (ROS) (97, 98). TNF-α is associated with MMP-1 and MMP-13, enzymes responsible for degrading the cartilage matrix (55, 56). OT also activates the JAK2/STAT1 pathway, participating in the degradation of Col II and the expression of MMP. In OA, OT activates the C-FOS pathway. C-FOS/AP-1 directly regulates MMP expression by binding to the AP-1 site (99). Roux CH et al. studied OA patients and observed that OT stimulates cartilage formation, increasing the expression of extracellular proteoglycans, collagen (Col) X, and chondrocyte matrix proteins (57). In summary, OT has broad anti-inflammatory, and antioxidant properties, and stimulates cartilage formation, protecting the cartilage matrix in OA (**Table 1**).

### Oxytocin and fracture

In addition to its potential therapeutic role in treating osteoporosis, OT has also shown potential in promoting the healing and repair of the pulp-dentin complex (100). Fracture healing is a complex process involving inflammation, healing, and remodeling. Endogenous OT has been found to have analgesic and anti-inflammatory effects in rats (101). Studies have revealed that OT accelerates fracture healing by enhancing the recruitment and differentiation of BMSCs (102). Furthermore, OT has been shown to improve the mechanical properties of healed bones, making them more resistant to future fractures. Following OT treatment, bones exhibit greater compressive loads, fewer pores, and larger trabecular bone volume (47).

This is the first study demonstrating that OT can induce bone formation in bone tissue engineering (29). Microporous b-TCP bone substitutes loaded with OT promote bone formation by regulating the RANKL/OPG ratio. Akay AS et al. mixed OT with poly (lactic-co-glycolic acid) and established a cranial defect model (103). They found that the OT carrier PLGA accelerated new bone formation in the first four weeks. OT stimulated a reduction in bone resorption, leading to a positive bone balance during the bone healing process (65).

OTR is present in OBs and OCs. OT phosphorylates OTR and activates the C-FOS pathway (39). This mechanism induces the differentiation of BMSCs into OBs to increase bone formation and regulates OCs to reduce bone resorption. Oxytocin also triggers the release of calcium ions (Ca2+) and activates the Ca2+-Erk1/2 pathway. Another mechanism involves the synthesis of nitric oxide (NO), regulating glycolysis and differentiation of OB. NO plays a crucial role in inhibiting bone resorption (104). Additionally, OT stimulates the secretion of PGE2 in the body (105). PGE2 can affect nerves and the environment, activate the EP4 receptor, and promote bone formation. PGE2 also regulates inflammation-related responses and inhibits the secretion of norepinephrine (NE) (106, 107). NE plays a significant role in bone metabolism, with two classical receptors, α-adrenergic receptor (α-AR), and β-adrenergic receptor (β-AR). NE can promote bone formation through α-AR and β1-AR and inhibit bone formation through β2-AR (70). *In vivo*, OT influences nerves and the environment, favoring increased new bone formation. Yuka Kato et al. demonstrated that OT is involved in the mechanism of dentin formation (108). Considering the impact of OT on dentin and bone formation, OT may become a future novel therapy (**Table 1**).

OT-based bone healing and repair therapies may have significant clinical implications. By accelerating the healing process and improving the quality of bones and muscles, OT can shorten recovery times and improve the prognosis of patients with fractures or those undergoing orthopedic surgery. This could have a significant impact on the quality of life of individuals affected by fractures, enabling them to regain mobility and functionality more quickly.

### Oxytocin and analgesia

In addition to its role in promoting bone formation, OT can also be used to alleviate postoperative pain in orthopedic surgery (109). OT can act as both a central and peripheral analgesic (110). Pain information is transmitted by nociceptive receptors, traveling through the spinal dorsal root ganglia to the spinal dorsal horn for initial integration, and then ascending via different pathways (spinothalamic tract, spinoreticular tract, and spinoparabrachial tract) to the cortical areas for advanced pain integration. Simultaneously, signals are conveyed to various midbrain structures for processing and integration of pain information, accompanied by parallel modulation from the pain descending inhibitory network formed by certain cortical areas, the hypothalamus, amygdala, and brainstem, ultimately resulting in “an adaptive aversive experience with sensory, emotional, cognitive, and social components.” Using viral vector-based chemical tracing and optogenetic techniques, it has been discovered that OT axonal projections and OTR expression occur in various structures associated with pain modulation, such as the superficial laminae of the spinal dorsal horn, cingulate cortex, hippocampus, central amygdala, periaqueductal gray, central nucleus of the amygdala, bed nucleus of the stria terminalis, ventral tegmental area, and periaqueductal gray or ventrolateral periaqueductal gray (111, 112).

Wahis et al. identified a specific subtype of astrocytes expressing OTR in the central amygdala CeL and demonstrated that OT directly acts on these astrocytes, affecting the emotional component of chronic pain (113). There is still controversy regarding the peripheral analgesic mechanism of OT, with the current mainstream view suggesting analgesia through the influence of OT-OTR on peripheral nociceptors and dorsal root ganglia (114). It has been confirmed that OTR is mainly expressed in the cell bodies or proximal dendrites of DRG neurons in the peripheral nervous system, rather than at axon terminals (**Table 1**). OTR is distributed in the cell membrane and nuclear subcellular structures of DRG cells, mainly in small-diameter DRG cells, indicating that OT may exert pain-modulating effects by activating OTR dynamically expressed in DRG neurons and directly utilizing primary nociceptive inputs from DRG (114, 115). Recently, the research team led by Li found that intravenous injection of OT in a paclitaxel-induced neuropathic pain model inhibits Nav1.7 currents by activating OT-mediated PKC phosphorylation on DRG neurons, thereby reducing excitability and pain transmission of DRG neurons to exert analgesic effects (116).

Li Xiaohua et al. discovered that OT selectively blocks presynaptic long-term potentiation, alleviating neuropathic pain and stress (117). Nasal administration of OT is currently the main route for pain relief (118). Additionally, OT can repair nerve damage (119). Following OT treatment, the transmission of light touch and nociceptive sensory nerves returns to normal (120). In bone cancer, OT alleviates bone cancer pain by inhibiting the upregulation of TLR4, TNFα, and IL-1β in the spinal cord (121). Furthermore, lower levels of OT are observed during the active phase of ankylosing spondylitis. The mechanisms by which OT influences ankylosing spondylitis are still under investigation.

## Conclusion

OT has been shown to improve myocardial hypertrophy, regulate blood pressure, exhibit antioxidant and anti-inflammatory properties, modulate energy metabolism, and alleviate anxiety, among other biological functions (122–124). OT and OTRs are widely distributed in the peripheral and central systems, including adipocytes, osteocytes, OBs, and OCs. OT, through specific binding to OTRs, acts on cells such as adipocytes, OBs, and OCs, upregulating osteogenic protein expression, inducing mineralization of OBs, inhibiting OCs, and ultimately promoting bone formation. Given its biological functions, OT may serve as a therapeutic approach for conditions such as OP, OA, and bone defects. Additionally, oxytocin can exert analgesic effects.

Currently, the therapeutic effects of OT on bone-related diseases are largely based on animal experiments, necessitating further clinical trials to assess the safety and efficacy of oxytocin-based treatments in patients. A major challenge is the need for targeted delivery systems to ensure effective and specific delivery of OT to target organs. Another limitation is the potential off-target effects of OT. Due to the presence of OTRs in various tissues throughout the body, including the cardiovascular system and the brain, there is a risk of systemic effects. Careful dose optimization and monitoring are required to minimize these side effects and ensure the safety of oxytocin-based treatments. Future research should elucidate the precise mechanisms by which OT promotes bone formation and determine the optimal dosage and administration route for oxytocin-based therapies.

In conclusion, OT plays a crucial role in bone metabolism. There is immense potential in the treatment of conditions such as OP, OA, and bone defects. By harnessing the power of OT, we may be able to improve bone health and alleviate the pain associated with bone disorders. OT is poised to become a novel therapeutic agent in the field of bone treatment.
